# Procalcitonin-to-Albumin Ratio vs. C-reactive Protein-to-Albumin Ratio in Predicting Sepsis and Its Severity in Children: A Prospective Observational Study

**DOI:** 10.7759/cureus.81935

**Published:** 2025-04-09

**Authors:** Vetsa Snigdha Hasa, Bandya Sahoo, Mukesh K Jain, Manas R Behera, Sibabratta Patnaik

**Affiliations:** 1 Pediatrics, Kalinga Institute of Medical Sciences, Bhubaneswar, IND; 2 Pediatric Medicine, Kalinga Institute of Medical Sciences, Bhubaneswar, IND; 3 Pediatric Intensive Care Unit, Kalinga Institute of Medical Sciences, Bhubaneswar, IND

**Keywords:** c-reactive protein, c-reactive protein to albumin ratio and pediatric sepsis, multi organ dysfunction syndrome, procalcitonin, procalcitonin to albumin ratio, serum albumin

## Abstract

Background: Sepsis is one of the leading causes of morbidity and mortality in children. The aim of our study was to compare the procalcitonin-to-albumin ratio (PAR) and C-reactive protein(CRP)-to-albumin ratio (CAR) in predicting sepsis and its severity in children and to know which one is better.

Methods: This prospective observational study was conducted in a tertiary care hospital from July 2022 to January 2024. Procalcitonin, CRP, and albumin, along with baseline investigations, were sent on the day of admission (day 0), day 2 of admission, and day 7 of admission for all the children with suspected sepsis.

Results: This study included 118 children diagnosed with sepsis who were analyzed. Among them, 83 (70%) were male. The median age was 11 years, with a range of two months to 17 years. The median duration of fever prior to hospitalization was four days. The mortality and morbidity distribution included total septic shock in 22 (19%), multiple organ dysfunction (MODS) in 12 (9%), and death in 13 (11%). In total, 22 (19 %) patients required inotropic support, and 15 (13 %) required respiratory support. The cut-off value for predicting sepsis obtained for PAR was 13.5 with sensitivity 49.47% and specificity 95.61% on the day of admission, whereas the cut-off value for CAR was 0.31 with sensitivity 67.36% and specificity 64.21% on the day of admission. Receiver-operating characteristic (ROC) curve analysis was done for the diagnostic value of PAR and CAR, and the area under the curve (AUC) for detecting sepsis were 0.73 (95%CI=0.634-0.827) and 0.67 (95% CI=0.544-0.796), respectively.

Conclusion: The study demonstrates that the PAR and CAR are valuable diagnostic markers for predicting sepsis in children. On the day of admission, PAR showed a higher specificity (95.61%) compared to CAR (64.21%), making it a reliable marker for ruling in sepsis, while CAR exhibited greater sensitivity (67.36%) for detecting cases. The ROC curve analysis revealed AUC values of 0.73 for PAR and 0.67 for CAR, indicating moderate diagnostic accuracy for both. These findings suggest that PAR and CAR can complement traditional markers in diagnosing pediatric sepsis and its severity, with PAR being particularly useful in identifying more severe cases. Further research is warranted to validate these markers and explore their prognostic utility over time.

## Introduction

Pediatric sepsis is one of the challenging and serious illnesses among the pediatric population. There are around 7.5 million deaths annually worldwide due to pediatric sepsis [1]. With prompt diagnosis and treatment, mortality can be reduced, and outcomes can be enhanced. Screening, diagnosis, judicious administration of antibiotics, and monitoring of response to antibiotics are made feasible by the existence of certain biomarkers. Further biomarkers can be useful in distinguishing between bacterial and viral infections [2]. While C-reactive protein (CRP) and procalcitonin (PCT) are the most commonly used biomarkers for diagnosing bacterial infections, both have several limitations. Although both have variable sensitivity and specificity, CRP has low accuracy as compared to PCT, which is a more specific biomarker of infection/inflammation and hence can aid clinicians in diagnosing sepsis [3].

Considering the fact that both PCT and serum albumin (ALB) are closely related to infection and inflammation, the PCT-to-ALB ratio (PAR) has been found to strongly correlate with mortality in adults with sepsis-induced acute kidney injury [4]. PAR is also an early diagnostic predictor in discriminating urosepsis from febrile urinary tract infections [5]. 

CRP and ALB are acute-phase proteins produced by the liver. In sepsis, CRP increases and positively correlates with the degree of infection [6], while ALB is often decreased. Wang et al. found the CRP-to-ALB ratio (CAR) serves as an effective indicator for early diagnosis of pediatric sepsis and had a higher value in combined diagnosis [7]. The presence of a standardized biomarker that is specific, sensitive, and effective assessment tool for early identification of sepsis in children would improve the outcome of pediatric sepsis [8]. It has been observed that a combination of biomarkers increases the sensitivity and specificity of tests rather than an individual biomarker [9,10]. Recent research studies show that higher PAR and CAR are independent predictors for neonatal sepsis [9,11]. There is much evidence to prove CRP and procalcitonin as independent markers for the diagnosis of sepsis in children [12-14]. However, there is no study combining PAR and CAR to increase the diagnostic accuracy for detecting pediatric sepsis and its severity. The common practice of using independent biomarkers for detecting sepsis has to be given a thought. In recent studies, PAR and CAR have been studied in different scenarios [5,9-11,15,16], but there are no studies regarding the CAR and PAR in correlation with pediatric sepsis in children. The main aim of our study was to analyze the role of PAR and CAR in predicting sepsis and its severity in the pediatric population.

## Materials and methods

This was a prospective observational study conducted in the Department of Paediatrics at a tertiary care hospital, Kalinga Institute of Medical Sciences (KIMS), Bhubaneswar, Odisha, India, over 18 months, from July 1, 2022, to January 1, 2024. The study aimed to evaluate the diagnostic and prognostic utility of biomarkers, specifically the PAR and CAR, in children with suspected sepsis. The study protocol was approved by the Institutional Ethical Committee of KIMS (approval number: KIMS/SLRC/PG/2022/79, dated June 24, 2022). Written informed consent was obtained from parents or guardians, and assent was taken from children aged ≥7 years who were capable of understanding the study. The study adhered to ethical guidelines, ensuring confidentiality and the rights of participants. 

Inclusion and exclusion criteria

*Inclusion Criteria* 

Children aged >1 month to 18 years admitted to the Pediatric Intensive Care Unit (PICU) with suspected clinical sepsis, as defined by the International Consensus on Pediatric Sepsis (2005), were included. According to this definition, sepsis is a systemic inflammatory response to infection that results in clinical signs and symptoms of infection along with the presence of Systemic Inflammatory Response Syndrome (SIRS).

Exclusion Criteria

Children with severe acute malnutrition, recent surgical interventions, primary liver diseases, nephrotic syndrome, or malabsorption syndromes were excluded to minimize confounding factors.

Sample size calculation

The sample size of 118 was determined based on a previous study by Li et al. [10], considering a 5% level of significance, 95% confidence interval, and 80% power.

Definitions

Criteria for Sepsis

Infection: There must be clinical evidence of an infection. This can be confirmed through either microbiological evidence (positive cultures), clinical signs (e.g., fever, chills, signs of pneumonia, meningitis, or urinary tract infection), or infectious focus (e.g., a documented source of infection).

SIRS: Two or more of the following must be present: Fever (temperature >38.0°C/100.4°F) or hypothermia (temperature <36.0°C/96.8°F, tachycardia (age-specific increased heart rate), tachypnea (age-specific increased respiratory rate) or need for mechanical ventilation, leukocytosis (WBC >12,000/µL), leukopenia (WBC <4,000/µL), or an increased band count (immature neutrophils >10%)

Severe Sepsis

Severe sepsis is sepsis complicated by organ dysfunction or tissue hypoperfusion. Evidence of organ dysfunction, which can include: (i) cardiovascular dysfunction: hypotension (low blood pressure), despite adequate fluid resuscitation, tachycardia (abnormally high heart rate), (ii) respiratory dysfunction: hypoxemia (low oxygen levels in blood), acute respiratory failure (need for mechanical ventilation), (iii) renal dysfunction: oliguria (urine output <1 mL/kg/hour) or anuria (no urine output), elevated serum creatinine or blood urea nitrogen (BUN), (iv) hematologic dysfunction: thrombocytopenia (low platelet count), disseminated intravascular coagulation (DIC), where blood clots form throughout the body, (v) metabolic acidosis: Lactic acidosis or elevated lactate levels indicating tissue hypo perfusion, and (vi) neurological dysfunction: altered mental status, confusion, or coma.

Septic Shock

Septic shock is severe sepsis with persistent hypotension despite adequate fluid resuscitation, leading to inadequate tissue perfusion and oxygenation, which can result in multi-organ failure and death. 

Data collection

Data were collected at three time points: on the day of admission (day 0), day 2 of admission, and day 7 of admission. Clinicodemographic information, including age, sex, and clinical parameters such as heart rate, respiratory rate, blood pressure, oxygen saturation, and temperature, were recorded. The need for respiratory support (e.g., nasal prongs, high-flow heated humidified nasal cannula, non-invasive ventilation, or mechanical ventilation) and circulatory support (e.g., inotropes) was documented. Sensorium was assessed using the Glasgow Coma Scale (GCS), and the pediatric Sequential Organ Failure Assessment (pSOFA) score was calculated to evaluate disease severity. 

Baseline investigations included complete blood count (CBC), renal function tests, and liver function tests. Biomarkers such as CRP, PCT, and serum albumin were measured on day 0, day 2, and day 7. Blood cultures were collected at admission to confirm sepsis. PAR and CAR were calculated to assess their diagnostic and prognostic relevance in sepsis. 

Statistical analysis

Data were analyzed using IBM SPSS Statistics for Windows, Version 28.0 (Released 2021; IBM Corp., Armonk, New York, United States). Continuous variables were expressed as median and range, while categorical variables were presented as frequencies and percentages. Receiver operating characteristic (ROC) curve analysis was performed to evaluate the diagnostic accuracy of PAR and CAR in predicting sepsis and disease severity. Youden’s index (sensitivity + specificity − 1) was used to determine the optimal cut-off values for PAR and CAR, balancing sensitivity and specificity for clinical utility. The area under the ROC curve (AUC) for both biomarkers was compared using Delong’s test. Diagnostic cut-off values, sensitivity, specificity, and 95% confidence intervals (CI) were calculated at a 5% significance level. 

Youden’s index provided a statistically robust method to identify optimal cut-off values, ensuring a balance between sensitivity and specificity for clinical decision-making. These biomarkers, along with ROC curve analysis, offered insights into their diagnostic and prognostic potential in pediatric sepsis.

## Results

This study included 118 children diagnosed with sepsis. Among them, 83 (70%) were male and 35 (30%) were female. The median age was 11 years, with a range of two months to 17 years. The median duration of fever prior to hospitalisation was four days (Table 1). The mortality and morbidity distribution included total septic shock in 22 (19%), multiple organ dysfunction (MODS) in 12 (9%), and death in 13 (11%). In total, 22 (19%) patients required inotropic support, and 15 (13%) required respiratory support.

**Table 1:** Demographic and laboratory values IQR: interquartile range; TLC: total leucocyte count; CRP: C-reactive protein; PCT: procalcitonin; ALB: albumin

Variables		Median (IQR)	Range
Age (years)		11 (10-14)	0.2-17
Duration of fever (in days) prior to hospitalization		4 (2-6)	1-30
Hemoglobin (g/dL)		9.6 (9-11.3)	4.5-21
TLC (10^3^/uL)		7 (4.5-19)	0.7-44
Total Bilirubin (mg/dL)		0.72 (0.6-0.9)	0.15-9.36
Creatinine (mg/dL)		0.64 (0.5-0.88)	0.1-3.9
CRP (mg/L)	Day		
	0	121 (35-220)	1.2-365
	2	120 (40-220)	0.5-402
	7	10 (10-50)	0.1-312
PCT (ng/ml)	Day		
	0	25 (3.2-45)	0.1-123
	2	27 (10-36.5)	0.2-125
	7	0.7 (0.5-5)	0.01-70
Albumin (g/dL)	Day		
	0	3.0 (2.7-3.3)	0.8-4.2
	2	3.0 (2.7-3.2)	1-4.0
	7	3.2 (3.2-3.5)	1.9-4.2
CRP/ALB ratio	Day		
	0	0.41 (0.17-0.85)	0.04-2.78
	2	0.37 (0.17-0.79)	0 -2.0
	7	0.03 (0.03-0.13)	0 -1.25
PCT/ALB ratio	Day		
	0	9.06 (5.63-18.33)	0.03-121.2
	2	8.77 (5.7-17)	0 -90
	7	0.21 (0.17-3.3)	0 -21.8

Diagnostic value of PAR and CAR in predicting sepsis

**Figure 1:**
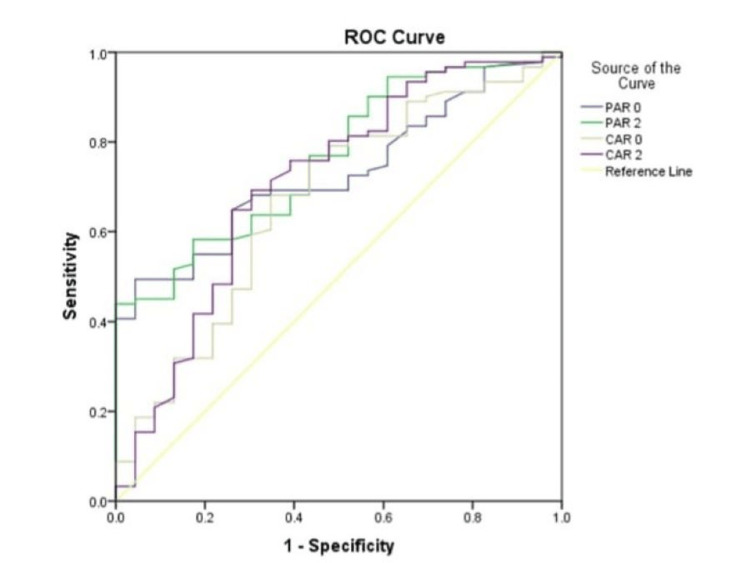
ROC curve analysis of CAR and PAR in predicting sepsis ROC: reciever operating characterstics; PAR: procalcitonin-to-albumin ratio; CAR: C-reactive protien-to-albumin ratio

PCT, CRP, and ALB tests were done on the day of admission, day 2, and day 7 of admission. The cut-off values taken for PCT and CRP were > 0.5 ng/mL and 5 mg/L, respectively. The normal value of ALB is 3.5-5 g/dL. The ROC curve analysis was done for the diagnostic value of PAR and CAR. As shown in Figure 1, AUC for detecting sepsis were 0.730 (95%CI: 0.634-0.827) and 0.670 (95%CI: 0.544-0.796), respectively, for PAR and CAR. The cut-off value obtained for PAR was 13.5, with a sensitivity of 49.47% and a specificity of 95.61% on the day of admission. The cut-off value for CAR was 0.31, with a sensitivity of 67.36% and a specificity of 64.21% on the day of admission. The diagnostic values of day 2 are described in Table 2.

**Table 2:** Diagnostic value of PAR and CAR in predicting sepsis PAR: procalcitonin-to-albumin ratio; CAR: C-reactive protein-to-albumin ratio Day 0: day of admission, Day 2: second hospital day

Test Result Variable(s)	AUC (95% CI)	Standard Error^s^	P Value	Cut-off	Sensitivity	Specificity	Youden Index
PAR Day 0	0.730 (.634-.827)	.049	.001	13.5	49.47	95.61	0.4508
PAR Day 2	0.764 (.666-.862)	.050	.000	13.22	44.21	100	0.4421
CAR Day 0	0.670 (.544-.796)	.064	.012	0.31	67.36	65.21	0.3257
CAR Day 2	0.709 (.582-.837)	.065	.002	0.33	64.21	73.91	0.3812

Predictive value of PAR and CAR in predicting severe sepsis

The cut-off value obtained for PAR was 16.37, with a sensitivity of 50% and a specificity of 78.8% on the day of admission. The cut-off value for CAR was 0.403, with a sensitivity of 69% and a specificity of 54.3% on the day of admission. The values of day 2 and day 7 of admission are given in Table 3.

**Table 3:** Diagnostic value of PAR and CAR for predicting severe sepsis PAR: procalcitonin-to-albumin ratio; CAR: C-reactive protein-to-albumin ratio PAR 0: PAR on the day of admission; PAR 2: PAR on day 2 of admission; PAR 7: PAR on day 7 of admission; CAR 0: CAR on the day of admission; CAR 2: CAR on day 2 of admission; CAR 7: CAR on day 7 of admission

Variables	AUC (95% CI)	P Value	Sensitivity	Specificity	Youden index	Cut off value
PAR -0	0.602 (0.473-0.731)	0.114	50	78.8	28.8	16.3
^PAR -2^	0.642 (0.519-0.764)	0.028	46.2	80.4	26.6	16.9
^PAR -7^	0.701 (0.588-0.813)	0.002	53.8	80.4	34.2	1.3
CAR- 0	0.606 (0.483-0.730)	0.098	69.200	54.3	23.5	0.403
CAR -2	0.701 (0.582-0.821)	0.002	57.700	80.4	38.1	0.632
CAR -7	0.599 (0.475-0.724)	0.123	80.800	40.2	21.0	0.028

**Figure 2:**
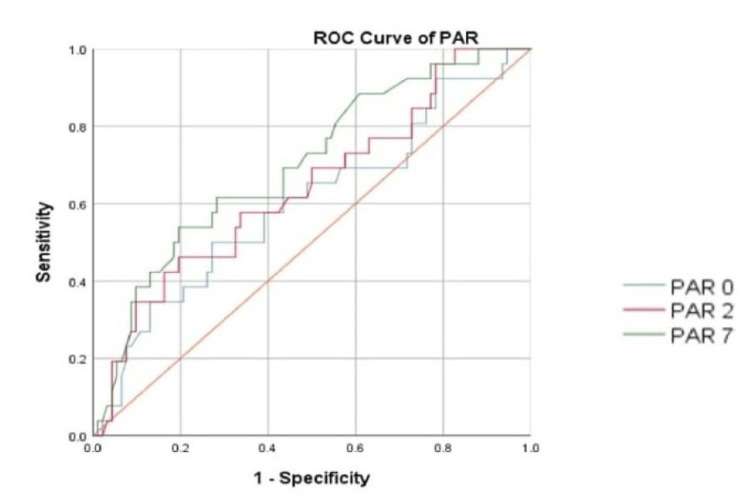
ROC of PAR in predicting severe sepsis PAR: procalcitonin-to-albumin ratio 0: day of admission; 2: day 2 of admission; 7: day 7 of admission

**Figure 3:**
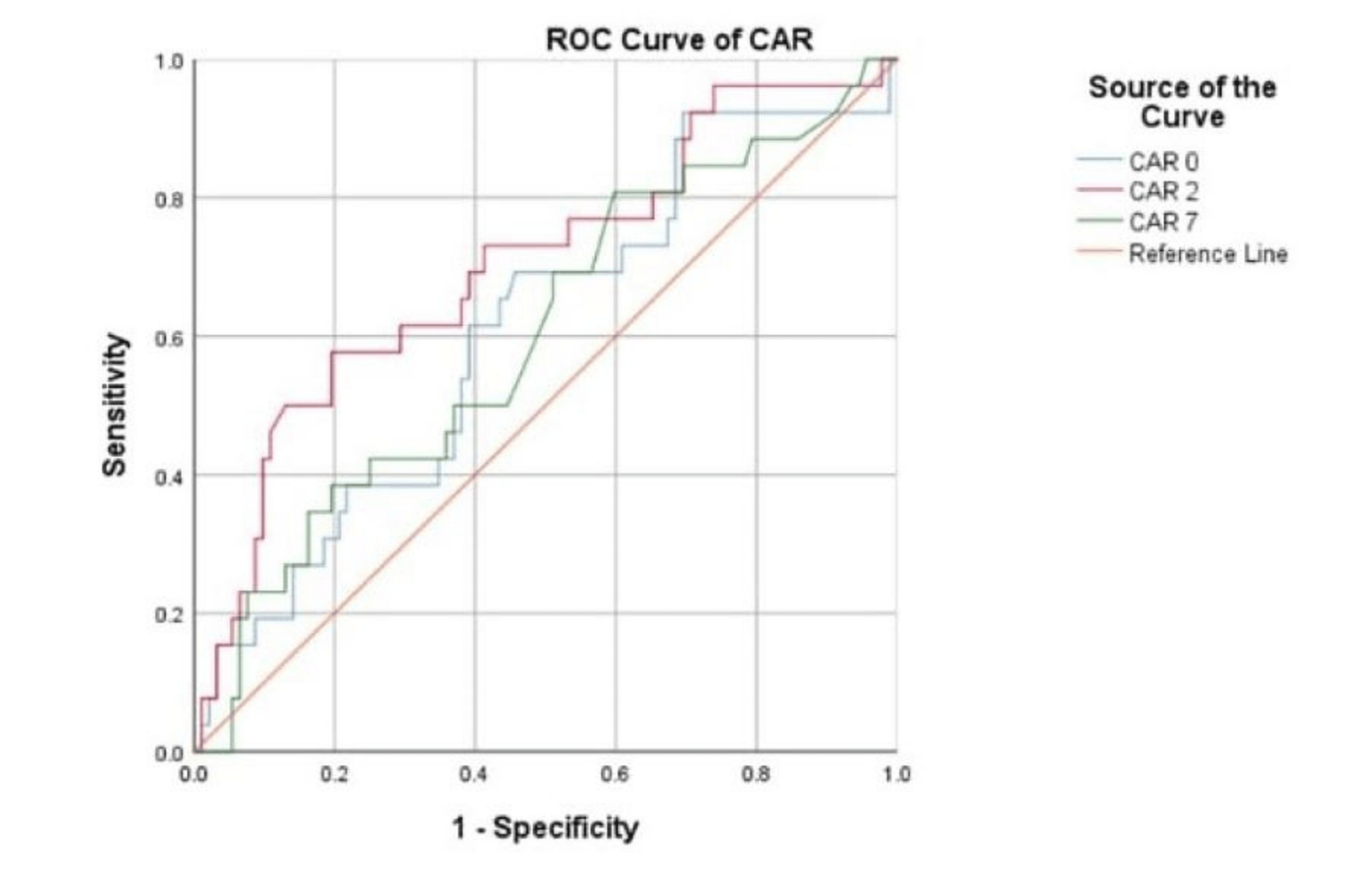
ROC of CAR in predicting severe sepsis CAR: C-reactive protien-to-albumin ratio 0: day of admission; 2: day 2 of admission; 7: day 7 of admission

The prediction of severe sepsis was assessed using AUC. As shown in Figures 2, 3, the AUC of PAR and CAR for predicting severe sepsis are 0.602 (95%CI: 0.473-0.731) and 0.606 (95%CI: 0.483-0.730), respectively. 

## Discussion

Early diagnosis of pediatric sepsis is of utmost importance for timely management and good outcome. In this cohort, we have studied the role of combined biomarkers like PAR and CAR for detecting sepsis and its severity in the pediatric population.

In our study, CAR was significant in predicting sepsis and also the severity of sepsis on day 2 of admission. The optimal cut-off value for CAR on admission was 0.31 (sensitivity 67.3% and specificity 65.2%), and day 2 of admission was 0.33 (sensitivity 64.2% and specificity 73.9%). According to a study by Wang et al., the clinical value of CAR in diagnosing pediatric sepsis revealed AUC of 0.809, sensitivity of 68% and specificity of 95% with cut-off of 0.41 [17], which is similar to our study.

In our study, the ROC curve analysis of CAR showed an AUC of 0.709 (p-value < 0.05) for predicting sepsis and an AUC of 0.701 (p-value <0.05) for severe sepsis on day 2 of admission, which shows a good discriminatory ability in predicting pediatric sepsis. A similar finding was established by Li et al. in predicting neonatal sepsis (AUC=0.74, p-value <0.01) and its severity (AUC=0.70, p-value < 0.001) [10].

The diagnostic efficacy of PCT in sepsis is well established; hence, we have combined ALB with PCT to recognize the superior diagnostic performance between the two. In our study, the diagnostic performance of PAR showed a cut-off value of 13.5 on the day of admission (sensitivity 49.4% and specificity 95.6%) and 13.22 on day 2 of admission (sensitivity 44.7% and specificity 100%). According to Li et al., there was a strong correlation between PAR with neonatal SOFA score and prolonged length of hospital stay in neonatal sepsis [11]. The cut-off value in their study was more than equal to 0.065 with sensitivity 64%, specificity 72%, AUC 0.72, and p-value <0.001 for predicting neonatal sepsis. However, in our study, the ROC curve analysis of PAR in predicting sepsis shows an AUC of 0.730 on the day of admission and an AUC of 0.764 on day 2 of admission.

We have also derived a correlation of PAR with sepsis severity. PAR was significant in detecting sepsis severity on day 2 and day 7 of admission. The cut-off value for predicting sepsis severity on day 2 of admission was 16.97 with sensitivity of 46% and specificity 80%, which is statistically significant with a p-value of 0.02; hence, our study shows higher PAR value is associated with the presence of sepsis and its severity.

There is no study of PAR in correlation with pediatric sepsis and its severity. However, a study by Ho et al. analyzes the 28-day mortality prediction in pediatric sepsis [18]. This was a retrospective study comparing the usefulness of lactate/ALB ratio, CAR and PAR in which the ROC curve analysis for 28-day mortality showed an AUC of 0.533 and cut-off value of 4.15 (sensitivity 60%, specificity 46%).

The novelty of this study lies in the analysis of the role of CAR and PAR in pediatric sepsis and also the comparison between them to find out which has a superior diagnostic value. This dual approach allowed us to assess not only the individual contributions of biomarkers but also their combined diagnostic efficacy, shedding light on their potential clinical utility. 

We determined the optimal cut-off values specific to this study population, which is one of the limitations of our study. Moreover, our study was constrained by a small sample size, and the cost burden was heightened due to the necessity of conducting tests three times. Furthermore, our study was conducted at a single center, potentially limiting the generalizability of our findings to the specific geographical area.

## Conclusions

The study demonstrates that the PAR and CAR are valuable diagnostic markers for predicting sepsis in children. On the day of admission, PAR showed a higher specificity compared to CAR, making it a reliable marker for ruling in sepsis, while CAR exhibited greater sensitivity for detecting cases. The ROC curve analysis indicated moderate diagnostic accuracy for both. These findings suggest that PAR and CAR can complement traditional markers in diagnosing pediatric sepsis and its severity and help in reducing sepsis-related mortality with PAR being particularly useful in identifying more severe cases. Further research is warranted to validate these markers and explore their prognostic utility over time.
